# The Roles of circRNAs in Liver Cancer Immunity

**DOI:** 10.3389/fonc.2020.598464

**Published:** 2021-02-04

**Authors:** Ying Tang, Mei Jiang, Hai-Mei Jiang, Zeng Jie Ye, Yu-Sheng Huang, Xiu-Shen Li, Bin-Yu Qin, Rui-Sheng Zhou, Hua-Feng Pan, Da-Yong Zheng

**Affiliations:** ^1^ Department of Oncology, Institute of Tumor, Guangzhou University of Chinese Medicine, Guangzhou, China; ^2^ Department of Oncology, Lingnan Medical Research Center of Guangzhou University of Chinese Medicine, Guangzhou, China; ^3^ Department of Oncology, Guangzhou University of Chinese Medicine, Guangzhou, China; ^4^ Department of Oncology, The First Affiliated Hospital of Guangzhou University of Chinese Medicine, Guangzhou University of Chinese Medicine, Guangzhou, China; ^5^ Department of Hepatopancreatobiliary, Cancer Center, Southern Medical University, Guangzhou, China; ^6^ Department of Hepatology, TCM-Integrated Hospital of Southern Medical University, Guangzhou, China

**Keywords:** liver cancer, circRNA, immune evasion, natural killer (NK) cells, innate immunity

## Abstract

Circular RNAs (circRNAs) are stable covalently closed non-coding RNAs (ncRNAs). Many studies indicate that circRNAs are involved in the pathological and physiological processes of liver cancer. However, the functions of circRNAs in liver cancer immunity are less known. In this review, we summarized the functions of circRNAs in liver cancer, including proliferative, metastasis and apoptosis, liver cancer stemness, cell cycle, immune evasion, glycolysis, angiogenesis, drug resistance/sensitizer, and senescence. Immune escape is considered to be one of the hallmarks of cancer development, and circRNA participates in the immune escape of liver cancer cells by regulating natural killer (NK) cell function. CircRNAs may provide new ideas for immunotherapy in liver cancer.

## Introduction

Liver cancer, a disease with high mortality and poor prognosis, is one of the most common malignant tumors in the world (1). Statistics show that liver cancer ranks the fifth in cancer incidence, the second in all cancer deaths, and the third in cancer mortality (2). Liver cancer includes three major pathological types: hepatocellular carcinoma (HCC), intrahepatic cholangiocarcinoma (ICC), and HCC-ICC mixed type (3). The occurrence of liver cancer is closely related to hepatitis B, hepatitis C, and non-alcoholic fatty liver disease (4–6). According to the patient’s overall conditions, a range of therapies have been utilized in the liver cancer treatment, such as surgical resection, liver transplantation, immunotherapy, local ablative therapies, and systemic chemotherapy. However, liver cancer is generally detected at the late stage because the patients might not perform the clinical symptoms at the beginning. Its recurrence is approximately 50–80% after treatment within 5 years (7). A better understanding of the molecular mechanisms of liver cancer is essential to largely improve the overall prognosis and discover novel effective therapies of liver cancer.

Immune escape refers to the growth and metastasis of tumor cells through various mechanisms to avoid recognition and attack by the immune system (8). The mechanisms of immune escape are mainly related to modifications, changes in tumor cells and alterations in the tumor immune microenvironment. Through the mechanisms of modification and change, tumor cells themselves can enhance their ability to evade immune surveillance and attack. Tumor has a highly heterogeneous structure, and tumor cells interact with many cells and factors including immune cells and immune factors to form a complex tumor immune microenvironment. The tumor microenvironment is the place where the immune system interacts with tumor cells. Natural killer (NK) cells are involved in tumor immune escape through multiple mechanisms (9–11). Various soluble factors and cytokines released by tumor cells or the tumor microenvironment reduce the activity of NK cells and their cytotoxic activity (12–14). Therefore, restoration of NK cell function is an important area of research in antitumor immunotherapy. Various strategies have been developed to restore NK cell function, including cytokine therapies, monoclonal antibodies, and adoptive cell transfer (15–18). NK cells can be divided into CD56bright and CD56dim based on the expression of CD56. Two subpopulations of CD56 are present, of which the CD56bright subpopulation can be amplified by IL-2 stimulation. The CD56bright subpopulation can be amplified by IL-2 stimulation, and about 10% of them express killer cells. Immunoglobulin-like receptor secretes synthetic TNF-associated apoptosis-inducing ligand (TRAIL). CD56dim subpopulation is insensitive to IL-2 stimulation, and 85% of CD56dim are KIR+ (19–22). In HCC, Rae1 is expressed on the surface of HCC cells, and this factor, as a ligand of NKG2D, the NK cell activation receptor, can activate NK cells and promote their anti-tumor immunity. On the other hand, the immune function of NK cells is limited, and the subsets of CD56dimNK cells in the peripheral blood of HCC patients were significantly lower than those in the healthy control group (23). CD56dimNK cells in the tumor area of HCC patients expressed fewer IFN-*γ* than non-CD56dimNK cells, which was associated with CD4+CD25+Tregs *in vitro*. During hepatocarcinogenesis, changes in the microenvironment of the extracellular matrix and the secretion of TGF-*β* by hepatic stellate cells can inhibit the activity and function of NK cells, thus weakening their monitoring function of hepatocytes (24). TGF-*β* secreted by Treg can inhibit NK cell activation by down-regulating NKG2D, affecting its immune killing function against liver cancer cells (25). Studies have reported that in liver cancer, circular RNAs (circRNAs) are involved in NK cell-associated immune evasion. Targeting circRNAs to restore NK cell function may provide new directions for the treatment of liver cancer.

Non-coding RNAs (ncRNAs), without the ability to translate into protein, were seen as ‘junk DNA’ by scientists for years. However, an array of ncRNAs nowadays has been discovered based on advances in sequencing technologies. In addition, accumulating lines of evidence indicate that ncRNAs play major roles in the processes of carcinoma initiation, progression, and metastasis by regulating proliferation, apoptosis, and cell cycle (26, 27). Based on the length and shape of RNA molecules, the ncRNAs are divided into three types including short ncRNAs (<200 nucleotides) and long ncRNAs (lncRNAs, >200 nucleotides) and circRNAs. CircRNAs, a novel category of endogenous ncRNAs, come from non-canonical back-splicing events of precursor mRNAs (pre-mRNAs) (28). CircRNAs were originally discovered in an RNA virus in 1976 and observed in eukaryotic cells in 1979 (29–31). CircRNAs have been recognized as ‘splicing noise’ or aberrant byproducts for a long time because they present a covalently joined continuous loop structure without 5′caps and 3′tails (32, 33). However, high-throughput sequencing and bioinformatics algorithms have clearly shown that circRNAs are not the accidental byproducts (34–37). Besides, circRNAs have been proved to be abundant and evolutionarily conserved, and are expressed in different types of tumors (38, 39). CircRNAs can not only regulate the expression of host genes by acting as transcriptional regulators, but also serve as microRNA (miRNA) sponge to fine-tune the regulatory axis of miRNA-mRNA (40–45). It has been confirmed that circRNAs can be used as prognostic biomarkers because they have remarkably stable characteristics (46). Furthermore, studies demonstrated that circRNAs can encode hidden peptides, and serve as a new drug targets resource bank (47–50). We herein illustrated the circRNAs molecular mechanisms connected to liver cancer, offered a novel perspective and a new horizon for cancer treatment and diagnosis. CircRNAs provide new ideas for the study of immune escape in liver cancer.

## Biogenesis of CircRNAs

**Figure 1:**
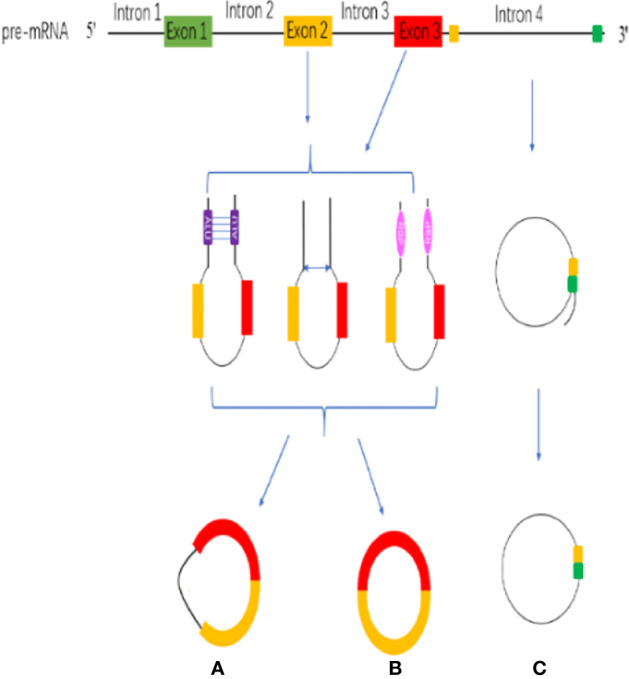
CircRNA biogenesis. **(A)** exon–intron circRNAs (EIcircRNAs). **(B)** exon circRNAs (ecircRNAs). **(C)** Intronic RNAs (ciRNAs).

CircRNAs are stable RNAs that are resistant to RNase R, circRNAs are mainly produced by the pre-mRNA through backsplicing. Although backsplicing is considered as an alternative splicing, it has different molecular mechanisms from linear alternative splicing. The hypothesis of backsplicing is that the downstream splicing site is reversed, and the upstream splicing site is connected to form a closed circRNA molecule. According to the region of origin, circRNAs can be divided into three types: (a) exon–intron circRNAs (EIcircRNAs), (b) exon circRNAs (ecircRNAs), (c) Intronic RNAs (ciRNAs) (**Figure 1**) (51).

The circularization model of circRNA is divided into intron circularization and exon circularization. There are three models for the circularization of EIcircRNAs and ecircRNAs: Intron pairing, Lariat and RNA-binding protein (RBP) (**Figure 1**) (52). Intron pairing-driven circularization, which known as direct backsplicing, is achieved by direct base pairs of intron flanking complementary sequences or reverse repeats (53, 54). The main component of intron pairing-driven circularization is the cis-acting elements, which enable direct base pairing between flank introns, either as short interspersed nuclear elements or as non-repeating complementary sequences (55, 56). Lariat-driven circularization, which is known as exon-skipping, is formed during linear splicing. During the transcription, the pre-mRNA can be partially folded, which formed an RNA lariat containing a 7 nt GU-rich element adjacent to the 5′ splice site and an 11 nt C-rich element closed to the branch point site consensus motif (28, 57). In addition, the third pattern is RBP-driven circularization. Through protein-protein interactions or the dimerization of the RBPs, the splicing sites are pulled closer, and the spliceosomes participate in the backsplicing reaction (40, 58). RBP-driven circularization is guided by two flanking intron pairs that are close to the flanking intron reverse complementary sequences (59). Above all, biogenesis of circRNA is a complicated process, and there are many regulatory details need to dig into.

## Functions of CircRNAs

**Figure 2:**
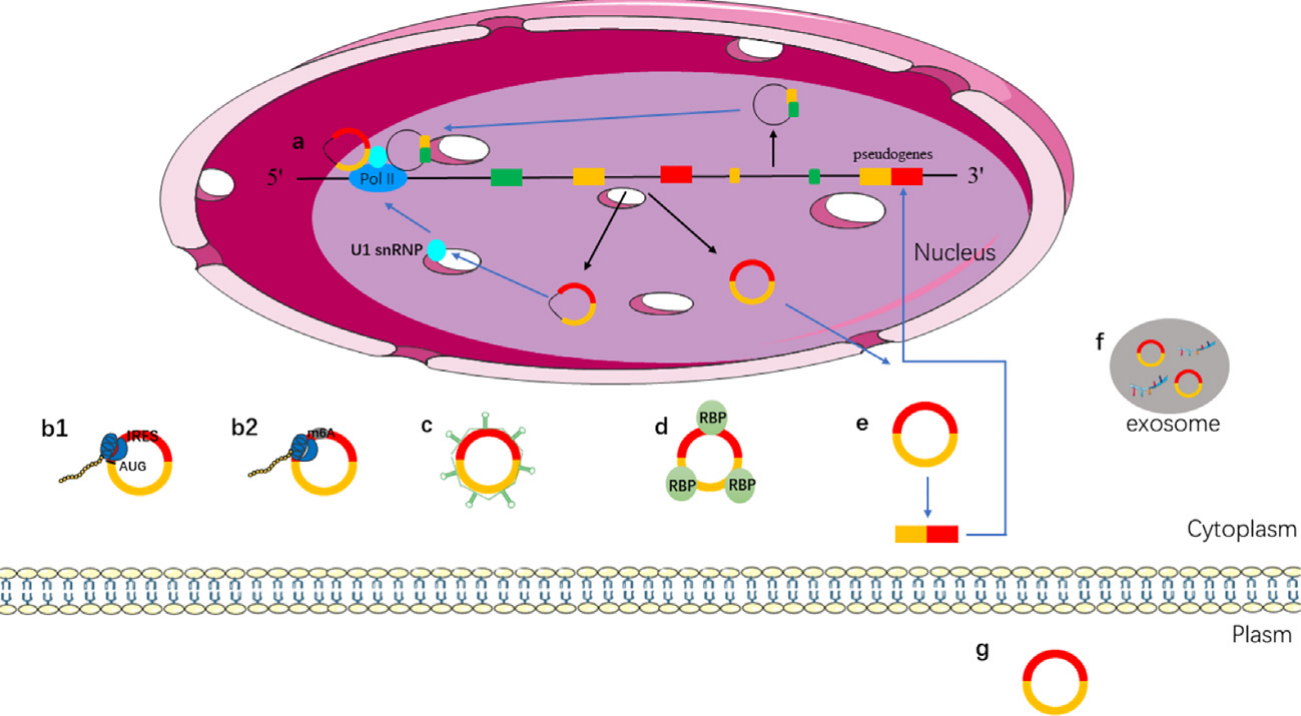
Functions of circRNAs. **(a)** EIcircRNAs and ciRNAs can regulate gene transcription *via* binding to U1 snRNP and RNA pol II in the nucleus; **(b)** ecircRNAs exported into cytoplasm; **(b1)** IRES-mediated cap-independent translation of ecircRNAs; **(b2)** m6A-mediated cap-independent translation of ecircRNAs; **(c)** ecircRNAs act as miRNA sponges; **(d)** ecircRNAs interact with RBP motifs; **(e)** ecircRNAs can form pseudogenes; **(f)** ecircRNAs can be secreted from the cell to outside *via* exosomes; **(g)** ecircRNAs act as biomarkers.

CircRNAs have become a hot topic in the field of ncRNA. The function of circRNAs has been extensively studied. Different types of circRNAs have different characteristics. EIcircRNAs and ciRNAs are usually located in the nucleus, and ecircRNAs are usually located in the cytoplasm. Different locations make them play different functions. The main mechanisms and biological functions of circRNAs are shown in **Figure 2** and discussed below.

## Transcription Regulation

A growing number of studies have shown that circRNAs play a role in regulating gene expression. CircRNAs, ciRNA, and EIciRNAs are located in the nucleus that can regulate protein expression by regulating transcription or post-transcription (59, 60). EIciRNAs can regulate transcription because they retain intronic sequences of host genes (61). For instance, circEIF3J and circPAIP2, which are located in the nucleus, can interact with U1 small nuclear ribosomal nucleoprotein (snRNP) to promote the transcription of host genes by binding to RNA polymerase II (RNA pol II) (41). Although EIciRNA and ciRNA do not have the function of miRNA sponge, they can regulate gene transcription and expression in transcription or post-transcription (**Figure 2a**).

## Translation

Traditionally, the 5′ and 3′ untranslated regions (UTRs) have been regarded as the basic elements of translation in eukaryotic cells. Although circRNAs contain exons, the absence of a 5′ cap structure and a poly A tail is considered to be ncRNA that does not encode proteins. However, an increasing number of researches have shown that some circRNAs can be translated into proteins. The researchers constructed artificial circRNAs containing an infinite reading frame to recruit 40s ribosomal subunits and translation into peptides *in vitro* (62). In 2017, Legnini et al. found that circ-ZNF609, a backsplicing product of ZNF609 exon 2, can be translated into proteins based on high-throughput phenotype screening. And, it can be translated into proteins in a splice-dependent and cap-independent manner (63).

More and more circRNAs are found to be able to translate into proteins, so how do the circRNAs initiate the translation mechanism? In some conditions, such as viral infection, mRNA translation can be initiated *via* internal ribosome entry site (IRES), which is an alternative mechanism for cap-independent translation (64, 65). IRESs can recruit ribosomes directly to initiate translation. IRES mediated translation is a widely accepted mechanism for initiating translation of circRNAs (66–68). Studies have shown that circRNA can be translated when an IRES is introduced into it (62). Both IRES and N6-methyladenosines (m6A) can drive circRNA translation (**Figure 2b**). The presence of methylated adenosine residues in m6A form is another cap-independent translation mechanism (69). Studies have shown that m6A can directly bind to eukaryotic initiation factor 3 and initiate the translation of circRNAs into proteins in human cells (69, 70).

## MiRNA Sponge

MiRNA is a type of ncRNA with a length of about 19–25 nt, which regulates the transcription of the target gene by binding to the 3′ UTR of the target gene through its seed sequence (71). Studies have shown that circRNAs contain miRNA response elements (MREs), which can competitively bind to miRNA (72). That is, circRNAs can bind to miRNA as miRNA sponges and then regulate the expression of target genes (71), such as, overexpression of circITCH can bind miR-17 and miR-224 to regulate p21 and PTEN genes to inhibit the development of breast cancer (73). CircHIPK3 inhibits the growth of cancer cells by binding to various miRNAs such as the tumor suppressor miR-124 (74, 75) (**Figure 2c**).

## Protein Regulation

Some circRNAs have been shown to bind to RBP, and can isolate RBP and transfer proteins to specific subcellular sites (76). The combination of circPABPN1 and RBP (HuR) prevented the interaction between HuR and PABPN1 (mRNA) and inhibited the translation of PABPN1 (77). High expression of circANRIL can be combined with peccadillo ribosomal biogenesis factor 1 (PES1) to control ribosomal RNA maturation (78). CircAmotl1 can promote the nuclear translocation of PDK1, AKT1, STAT3, c-myc, and other proteins by interacting with RBP and regulate the expression of corresponding target genes (79–81). The above lines of evidence suggest that circRNAs can regulate the function of proteins by binding to PBP instead of a single protein (**Figure 2d**).

## Form Pseudogenes

Pseudogenes are typically derived from reverse-transcriptional of linear mRNA, which integration into the host genome. In the human genome, thousands of pseudogenes are found at about 10% of the gene sites (82, 83). In 2016, the research revealed for the first time that mammalian genomes contain pseudogenes derived from circRNA by establishing a new type of computing analysis process (CIRCpseudo). It revealed that mice circSATB1 source of pseudogenes can be combined with CTCF, which prompts the pseudogenes derived from circRNAs to have the potential to control gene expression. This study showed a fresh perspective on the fact that circRNAs can be inserted into the genome *via* reverse transcription to alter genomic genetic information and regulate gene expression. Furthermore, many pseudogenes derived from circRNAs have been identified by searching for non-collinear backsplicing in both mouse and human genomes (84). In mice, the reverse transcription of circRFWD2 produced pseudogenes associated with long terminal repeats. The molecular mechanism of circRNA reverse transcription remains to be further studied (**Figure 2e**).

## Other Functions

Exosomes are a type of vesicles with a diameter of 40–150 nm; it is released by the majority of cell types (85). Exosome contains miRNA, lncRNA, circRNA, mRNA, transcription factors, lipids, and proteins (86). Exosomes can be used for liquid biopsy to monitor the development and metastasis of tumors. CircRNAs can be transported to the extracellular *via* exosomes (**Figure 2f**) (87). It has been found that exosomes contain abundant circRNAs, and the role of exosomal circRNAs remains to be further explored. CircRNA can be secreted into the blood, saliva, and other body fluids as a biomarker for disease prediction (**Figure 2g**). CircRNA is stable in body fluids because of its properties, and it is a promising biomarker for the diagnosis of cancer (88).

## The Functions of CircRNAs in Liver Cancer

**Figure 3:**
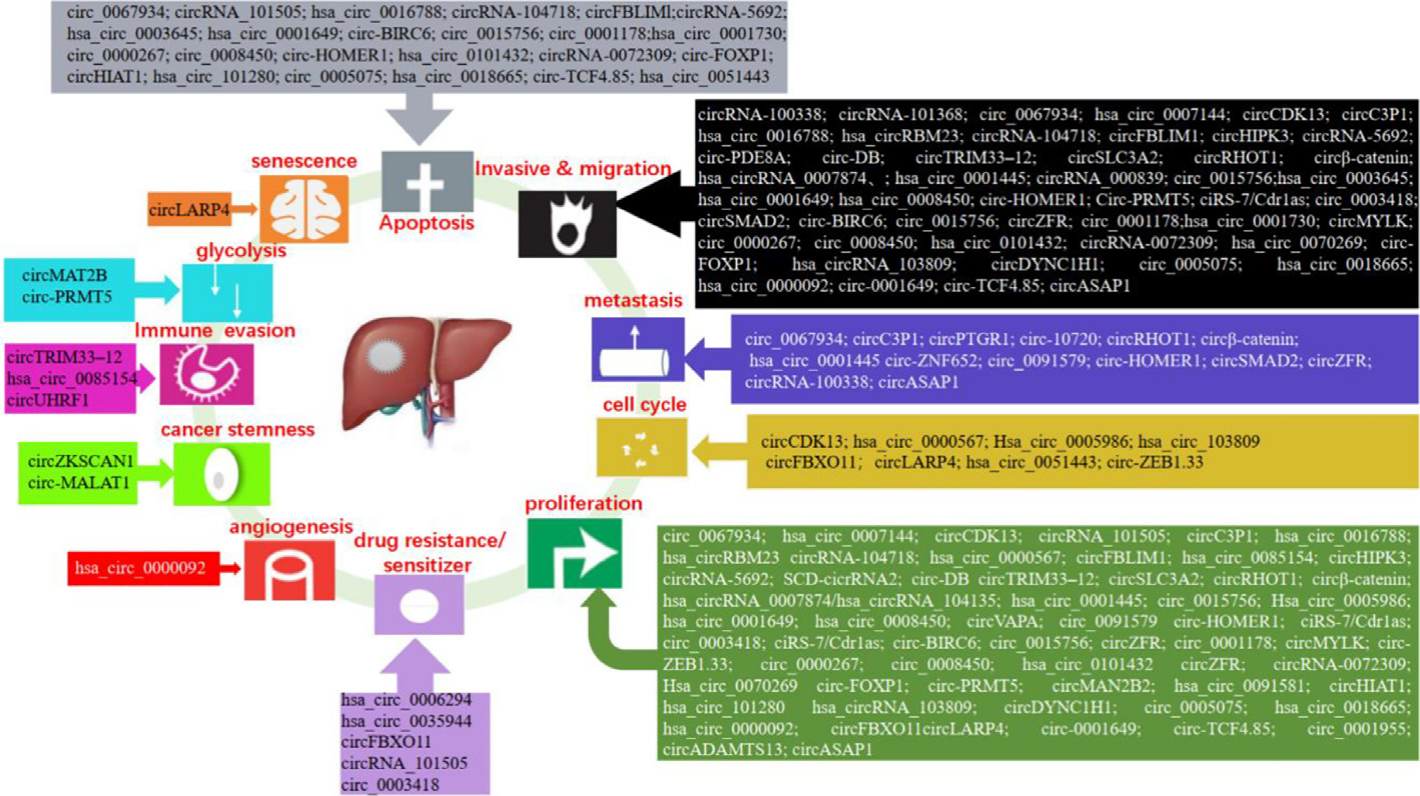
Functions of circRNAs in liver cancer.

The high mortality, poor prognosis, and lack of effective treatment methods of liver cancer force us to search for effective therapeutic targets and better tumor biomarkers. The studies have shown that a large number of circRNAs are abnormally expressed in liver cancer, which play a regulatory role in the development of liver cancer. The expression and function of circRNA in liver cancer are shown in **Figure 3**.

## Proliferative, Metastasis, and Apoptosis

Studies have shown that circRNAs can regulate the proliferation, migration, invasion, apoptosis, and metastasis of liver cancer cells. In liver cancer, hsa_circ_0000567, hsa_circ_0085154, hsa_circRNA_0007874, hsa_circ_0005986, hsa_circ_0001730, circRNA-0072309, hsa_circ_0070269, circHIAT1, circADAMTS13, ciRS-7/Cdr1as, and hsa_circ_0018665 suppressed cell proliferation (44, 89–98). While, hsa_circ_0101432, SCD-circRNA 2, circVAPA, circ_0015756, circ_0001178, circMYLK, circ-ZEB1.33, circZFR, circ-FOXP1, circMAN2B2, hsa_circ_0091581, hsa_circ_0005075, hsa_circ_101280, hsa_circ_103809, circDYNC1H1, hsa_circ_0000092, circFBXO11, circLARP4, hsa_circ_0001649, circ_0001955, and circ-TCF4.85 promoted tumor growth (99–120). Circ_0067934, hsa_circ_0007144, hsa_circRBM23, circHIPK3, circSLC3A2, circRHOT1, circβ-Catenin, circRNA-104718, circ-PRMT5, ciRS-7/Cdr1as, exosomal circ-DB, circ_0015756, circ_0091579, and circZFR enhanced cell proliferation, migration, and invasion (121–134). However, circCDK13, circC3P1, circ_0003418, circTRIM33-12, hsa_circ_0001445, and hsa_circ_0008450 inhibited cell proliferation, migration, and invasion (135–140). The study showed that circRNA_100338 increased cell invasive (141). CircRNA_000839 enhanced cell invasion and migration (142). Exosomal circPTGR1, circASAP1, and exosomal circRNA-100338 increased cell metastasis (143–145). Circ-10720 and circ-ZNF652 induced epithelial–mesenchymal transition (EMT) (146, 147). CircSMAD2 suppressed the EMT (148). And, circRNA_101368 suppressed the migration (149). The research showed that circRNA_101505 decreased cell proliferation and induced apoptosis (150). Conversely, hsa_circ_0016788, circFBLIM1, circ-BIRC6, circ_0000267, and circ_0008450 promoted cell proliferation, invasion, and suppressed the apoptosis (151–155). The research validated that circRNA_5692 suppressed the progression and invasion, induced apoptosis (156). On the contrary, hsa_circ_0003645 promoted cell migration, invasion and suppressed cell apoptosis (157). Circ-HOMER1 enhanced the proliferation, migration, invasion, and suppressed apoptosis (158). In addition, exosomal hsa_circ_0051443 enhanced cell apoptosis (159).

## Liver Cancer Stemness

Both cancer stem cells (CSCs) and circRNAs could affect the carcinogenesis and development of liver cancer, but there are few studies on the relationship between CSCs and circRNAs. Recent studies have found that circ-MALAT1, generated by the backsplicing of lncRNA, promoted the self-renewal of liver cancer CSCs (160). In addition, the researchers found that circZKSCAN1 can regulate the CSCs of HCC *via* Qki5/circZKSCAN1/FMRP/CCAR1/Wnt signaling axis (161). These findings revealed the role of circRNA in regulating stem cells and enrich the function of circRNA.

## Cell Cycle

An increasing number of studies have shown that circRNAs can be involved in the regulation of the cell cycle in liver cancer. For instance, hsa_circ_0000567 induced G1/S arrest in HCC cells by sponging miRNA-421 (89). Hsa_circ_0005986 suppressed the cell proliferation of HCC through promoting the G0/G1 to S phase transition (91). Circ-ZEB1.33 increased the percentage of S phase by regulating CDK6/Rb (105). Inhibition of hsa_circRNA_103809 significantly induced G1/S arrest (113). Down-regulation of circFBXO11 induced G1/G0 arrest (116). Furthermore, exosome-derived circRNA could be involved in the regulation of the cell cycle. Such as, exosome-transmitted hsa_circ_0051443 arrested the cell cycle in HCC (159).

## Glycolysis

Hepatoma cells required glycolysis to meet their proliferation needs under hypoxia conditions, and glucose reprogramming is a feature of cancers. Under the hypoxia environment, circMAT2B enhanced glycolysis of HCC *via* the miR-338-3p/PKM2 axis (162). Furthermore, circ-PRMT5 increased glycolysis of HCC by the miR-188-5p/HK2 axis (129).

## Angiogenesis

Cancer cells secrete the angiogenic factors that lead to the formation of abnormal vascular networks. Tumor blood vessel is the key target of tumor treatment. A recent study found that hsa_circ_0000092 promoted angiogenesis in HCC (115).

## Drug Resistance/Sensitizer

Resistance to chemotherapy is one of the causes of failure in the treatment of hepatocellular carcinoma. The research showed that circRNA_101505 inhibited cisplatin chemoresistance through miR103/Oxidored-Nitro Domain-Containing Protein 1 pathway (150). In addition, circ_0003418 sensitized HCC to cisplatin by Wnt/*β*-Catenin pathway (137). CircFBXO11 regulated oxaliplatin resistance through miR-605/FOXO3/ABCB1 axis in HCC (116). The expression of hsa_circ_0006294 and hsa_circ_0035944 was decreased in resistant HCC cells, and they may play a key role in sorafenib-resistant HCC cells (163). Therefore, circRNAs may provide us with a new strategy for the treatment of HCC.

## Senescence

Cell senescence is a defense mechanism to prevent and control cell damage and a barrier to prevent tumorigenesis. p53 and p21 are regulatory molecules in the senescence process. Research has found that circLARP4 promoted cellular senescence by regulating miR-761/RUNX3/p53/p21 signaling in HCC (117).

## Immune Evasion

Dysfunction of the immune system can lead to abnormal immune surveillance of liver cancer, and liver cancer cells can also act on the immune system to lead the immune escape. NK cells account for 50% of the total number of hepatic lymphocytes and are cytotoxic cells with antitumor functions mediated by the release of cytotoxic granules, FasL and TRAIL (164). NK cells do not rely on antigen presentation; this allows NK cells to target stress and damaged self-cells (165).

**Figure 4:**
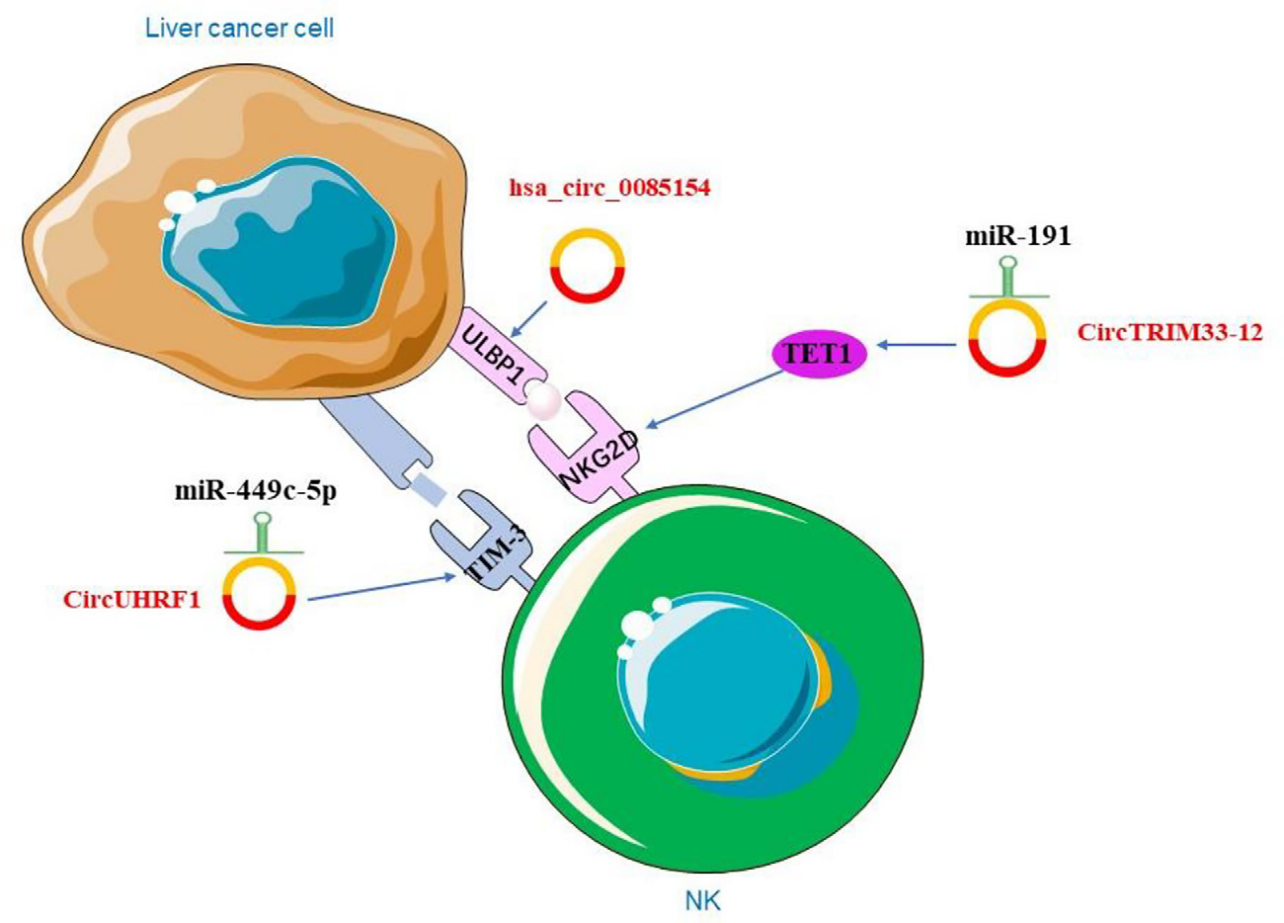
The role of circRNAs in liver cancer immunity.

Liver cancer cells avoid being destroyed by immune escape. Studies showed that circRNA could be involved in immune escape. Activation receptor natural killer group 2 member D (NKG2D) and its ligands in NK cells play a crucial role in cell-mediated immune responses to cancer (166). The researchers examined the expression of NKG2D in 200 patients with HCC and showed that the number of NKG2D-positive cells in HCC tissues was significantly reduced compared to adjacent non-tumor tissues. The expression of circTRIM33-12 was positively correlated with the number of NKG2D-positive cells in HCC. The result showed that circTRIM33-12 may enhance immune function by protecting Ten eleven translocation 1 (TET1) *via* sponging miR-191 (138). TET1, one of the 2-OG-dependent dioxygenases, is involved in regulating the formation of 5-hydroxymethylcytosine (5hmC) and has been proposed to be involved in DNA demethylation process (167). The studies have indirectly linked TET1 as a tumor suppressor in HCC (168). Hsa_circ_0085154 could enhance the innate immune monitoring effect of NK cells by up-regulating UL16 binding protein 1 (ULBP1), which suggests that circRNA may play a role in tumor immunity (169). In HCC, hsa_circ_0085154 promoted ULBP1 expression and assisted NK cells to recognize target tumor cells (169). ULBP1 is an NKG2D ligand that activates receptors expressed by NK cells (170) (**Figure 4**). NKG2D is a basic activation receptor belonging to the C-type lectin-like family that is constitutively expressed on NK cells (171). The apparently invariant activation receptor NKG2D binds promiscuously to a variety of ligands, such as major histocompatibility complex class I-associated chains A and B (MICA/B) and a unique family of long 16 binding proteins (ULBPs), which are poorly expressed on healthy cells, but they are up-regulated under DNA damage (172). The up-regulation of these ligands may lead to a shift in NK cell homeostasis from inhibition to activation. The research revealed that ULBP1, one of the NKG2D ligands, was not expressed in poorly differentiated human hepatoma tissues and cell lines, but was abundantly expressed in hyperplastic abnormal nodules and well to moderately differentiated HCC cells (172). These findings provided conclusive evidences for the role of NK cells and the NKG2D receptor pathway in immune surveillance of HCC. In addition, HCC-derived exosomal circUHRF1 induced impairment of IFN-*γ* and TNF-α secretion in NK cells. In HCC, high level of circUHRF1 suggested poor clinical prognosis and dysfunction of NK cells. CircUHRF1 inhibited the secretion of NK cell-derived IFN-*γ* and TNF-α. High level of plasma exosomal circUHRF1 was associated with a decreased proportion of NK cells and decreased NK cells tumor infiltration. In addition, circUHRF1 up-regulated the expression of T cell immunoglobulin and mucin domain 3 (TIM-3) by degrading miR-449c-5p, thereby inhibiting the function of NK cells (173) (**Figure 4**). TIM-3 plays an important role in cell immunity, it was expressed in NK cells and affects cellular immune responses (174). In recent years, many studies have focused on the expression of TIM-3 in HCC and its mechanism (175). TIM-3 polymorphisms have been found to play an important role in the susceptibility and characteristics of HCC. The TIM-3 promoter region is associated with certain features of HCC, including lymph node metastasis and tumor stage (176). Modulation of the role of TIM-3 in innate immunity offers new directions for HCC treatment.

## Conclusion

Structurally and mechanistically, the liver is an immune organ. It is rich in immune cells. Immune cells including dendritic cell (DC), NK cell, myeloid-derived suppressor cell (MDSC), CD8+ T cell, CD4 +T cell, regulatory T cell (Treg), T helper cell 1 (Th1), T helper cell 2 (Th2), T helper cell 17 (Th17), and tumor-associated macrophages (TAMs) (177–179). Liver cancer is one of the most common malignancies in the world. It is seriously threatening the health of Chinese people. In recent years, tumor immunotherapy is a major advance in cancer treatment, and targeted blocking of PD-1/PD-L1 immune checkpoints antibody-therapy is a milestone in the development of cancer immunotherapy. Currently, the FDA approved PD-1 antibody drug nivolumab (Opdivo) is being used in the treatment of cancer, and pembrolizumab (Keytruda) with the popular anti-cancer drug docetaxel (Sorafenib) combination for the treatment of HCC has been effective (180, 181). However, due to the primary/secondary drug resistance, immune escape, and antibody-drug effectiveness, the survival rate did not increase significantly in liver cancer. Therefore, it is of great significance to find a new way to improve the immunotherapy of liver cancer. NK cells are a new target for immunotherapy. There is a growing body of research using NK cell-related therapies to fight cancer (182, 183). NK cell-mediated immune surveillance is an important mechanism for tumor suppression. NK cells kill tumor cells through the release of perforin and granzyme and the secretion of pro-inflammatory cytokines and chemokines (184).

More and more studies have shown that circRNAs are involved in the carcinogenesis and progression of liver cancer. In this review, we summarized the functions of circRNAs in liver cancer. We found that circRNAs affect the cytotoxicity of NK cells. CircUHRF1 up-regulated TIM-3, the immune checkpoint, to inhibit the function of NK cells. Binding of TIM-3 to its ligand induces immune tolerance by depletes NK cells (185). It has been found that in tumor cells, immune checkpoints can lead to NK cell dysfunction, blocking these immune checkpoints (*e.g.* TIM-3, NKG2A, CTLA-4, PD-1, KIR2DL-1/2/3, CD96, TIGIT) can restore the function of NK cells (186). We can inhibit circUHRF1 to enhance NK cell function by down-regulating the expression of TIM-3. CircUHRF1 may provide a potential therapeutic strategy for immune checkpoints in liver cancer. More circRNAs regulating immune checkpoints are yet to be discovered, and targeting circRNAs provided a new direction for immune checkpoint therapy. Furthermore, circRNAs can affect the function of NK cells by regulating the receptor and ligand of NK cells. However, the relationship between circRNAs and other immune cells still needs further study. Understanding the mechanism of circRNAs in HCC patients is important in the design of effective immunotherapeutic protocols. Although circRNAs have shown an important role in liver cancer, many fields remain to be studied. For instance, the mechanism that ecircRNAs transported from the nucleus to the cytoplasm? the degradation of circRNAs?

## Author Contributions

YT and H-MJ wrote the draft of the manuscript. ZY and Y-SH collected the data. R-SZ contributed to the discussion. D-YZ and H-FP organized the structure of the manuscript. YT and D-YZ contributed to the conception of the work. X-SL and B-YQ contributed to the revision. All authors contributed to the article and approved the submitted version.

## Funding

This work was supported by the Project of Administration of Traditional Chinese Medicine of Guangdong Province of China (Project No. 20201109) and National Natural Science Foundation of China (Project No. 81973816).

## Conflict of Interest

The authors declare that the research was conducted in the absence of any commercial or financial relationships that could be construed as a potential conflict of interest.
